# Evaluation of a novel, multi-functional inhibitor compound for prevention of biofilm formation on carbon steel in marine environments

**DOI:** 10.1038/s41598-021-94827-9

**Published:** 2021-08-03

**Authors:** Benjamin Tuck, Elizabeth Watkin, Maria Forsyth, Anthony Somers, Mahdi Ghorbani, Laura L. Machuca

**Affiliations:** 1 Curtin Corrosion Centre, WA School of Mines: Minerals, Energy and Chemical Engineering, Curtin University, Kent Street, Bentley, WA 6102 Australia; 2 Curtin Medical School, Curtin University, Kent Street, Bentley, WA 6102 Australia; 3 Institute for Frontier Materials, Deakin University, Burwood, VIC 3217 Australia

**Keywords:** Microbiology techniques, Microscopy, Antimicrobials, Bacteria, Water microbiology, Metals and alloys, Ocean sciences

## Abstract

Chemical biocides remain the most effective mitigation strategy against microbiologically influenced corrosion (MIC), one of the costliest and most pervasive forms of corrosion in industry. However, toxicity and environmental concerns associated with these compounds are encouraging the development of more environmentally friendly MIC inhibitors. In this study, we evaluated the antimicrobial effect of a novel, multi-functional organic corrosion inhibitor (OCI) compound, cetrimonium trans-4-hydroxy-cinnamate (CTA-4OHcinn). Attachment of three bacterial strains, *Shewanella chilikensis, Pseudomonas balearica and Klebsiella pneumoniae* was evaluated on wet-ground (120 grit finish) and pre-oxidised carbon steel surfaces (AISI 1030), in the presence and absence of the new OCI compound. Our study revealed that all strains preferentially attached to pre-oxidised surfaces as indicated by confocal laser scanning microscopy, scanning electron microscopy and standard colony forming unit (CFU) quantification assays. The inhibitor compound at 10 mM demonstrated 100% reduction in *S. chilikensis* attachment independent of initial surface condition, while the other two strains were reduced by at least 99.7% of the original viable cell number. Our results demonstrate that CTA-4OHcinn is biocidal active and has promise as a multifunctional, environmentally sound MIC inhibitor for industrial applications.

## Introduction

Microbiologically influenced corrosion (MIC) is an electrochemical process leading to dissolution of a substratum, which is initiated, facilitated or maintained by microorganisms and their metabolisms^1–4^. MIC accounts for at least 20% of the total global corrosion cost ^5–7^ and including mitigation measures this figure is over $2.5 trillion annually^8^. Even if microorganisms are not directly corroding a substrate, biofouling is also a major concern leading to decreased efficiency of equipment such as heat exchangers and transport vessels^1^. The various mechanisms of MIC are governed by biofilm formation, which occurs when planktonic microorganisms colonise a surface, becoming sessile^9^. The term *biofilm* specifically pertains to aggregates of microorganisms suspended in a matrix comprised of extracellular polymeric substances (EPS) including but not limited to extracellular DNA (exDNA), proteins, carbohydrates and other trapped organic and inorganic compounds^10^. The difficulty in controlling MIC and biofouling is due in part to the flexibility and ubiquity of microorganisms in almost all environments on Earth^11–13^. Such widespread success is owed to the ability of biofilms to provide nutrients, protection and facilitate interspecies interactions, often giving rise to enhanced chemical and physical tolerance of microbial populations. To develop biofilm communities, planktonic cells must first undergo a series of attachment and adhesion stages^14^. Therefore, understanding microbial attachment and adhesion is of particular interest to MIC research.

Attachment of planktonic cells is the first reversible stage of biofilm development, eventually resulting in adhesion; the permanent irreversible association of a cell with the surface. These initial interactions with the surface are complex, specific and active processes, and there are still gaps in understanding of these interactions. In particular, limited work specifically evaluates bacterial attachment to steel surfaces. We know that steel grades have been associated with different rates of early attachment in some microorganisms; for example, Javed et al. point out that *Escherichia coli* attachment to carbon steel (CS) may be impacted by cell and substrate characteristics, including CS grade and pearlite content^15^. The authors concluded that *E. coli* exhibited preferential attachment to pearlite-free locations. In later attachment (> 60 min), ferrous ions have been associated with enhanced attachment to CS^16^, indicating attachment is a dynamic process affected by environmental conditions. Likewise, the presence of dissolved iron and precipitated iron oxides appear to have an effect on bacterial populations. Appenzeller et al. revealed iron presence provides growth advantages to *Escherichia coli* in drinking water^17^. Similarly, *Shewanella oneidensis* was found to respond to iron oxide surfaces in anaerobic conditions by a two to five times increased adhesion energy for electron transfer respiration^18^. On the same note, Kim et al. found attachment of *Enterococcus faecalis* was favoured at locations on activated carbon impregnated with iron oxides^19^. Although the affiliation of various bacterial strains with iron oxides has been established for decades, this phenomenon has not yet been investigated in carbon steel and associated iron corrosion products formed in seawater. Understanding the connection between metal oxides and microbial attachment to steel in seawater is a primary goal in understanding biofilm formation and its mitigation in marine structures.

Biocides and corrosion inhibitors (CI) are a primary method for controlling bacterial proliferation and corrosion in marine infrastructure. Biocides frequently employed by industry today include glutaraldehyde, tetrakis hydroxymethyl phosphonium sulphate (THPS), organo-sulphur compounds and quaternary ammonium compounds (QUATS), which often have derivatives or additives to improve biocidal function. More information can be found in a recent review by Suarez et al.^20^. Although effective, such compounds are toxic and environmentally hazardous leading to increased global stringency governing their application^21,22^. On the other hand, application of CIs alone without also controlling bacteria in the system may still lead to MIC once the CI has been degraded or depleted via biotic or abiotic mechanisms. Increasing regulations and awareness of the natural environment have resulted in a surge of interest in development and manufacture of novel environmentally sustainable compounds^21,23^. Likewise, the need to apply both biocides and CIs has resulted in efforts to combine the two compounds into a single multi-functional inhibitor compound. A prime example is the green biocidal organic corrosion inhibitor (OCI) *cetrimonium nalidixate*, a combination of cetrimonium bromide and nalidixic acid^21^. Seter and colleagues reported enhanced solubility and antimicrobial capacity of the multi-functional compound when compared to the individual compounds. The mechanism of corrosion inhibition is suggested as suppression of the anodic reaction by nalidixate, which is strengthened by the presence of cetrimonium. On the other hand, the two compounds used to produce cetrimonium nalidixate are antimicrobials employed in the pharmaceutical industry. Based on enhanced functional range and increased environmental sensitivity, multifunctional OCIs show great promise for the future of biocides and corrosion inhibition^24^.

For the present study, a novel multi-functional OCI was synthesised as described elsewhere^25^. The compound CTA-4OHcinn, is a quaternary ammonium carboxylate, which combines the organic *trans*-4-hydroxy-cinnamate anion with hexadecyl trimethyl ammonium cation. An established corrosion inhibition capacity exists for the organic anion through the formation of wormlike micelles, which attach to the CS substrate, forming a bilayer at the interface^25^. Additionally, the cation is a known antimicrobial surfactant^21^. Combined, it is suggested that this compound forms a layer on the CS substrate exhibiting effective corrosion inhibition while also inhibiting microbial attachment. Since CTA-4OHcinn has previously demonstrated effective corrosion inhibition, the purpose of this study is to evaluate the capacity of this OCI to reduce attachment of three bacterial isolates, therefore preventing biofilm formation. *Shewanella chilikensis, Pseudomonas balearica* and *Klebsiella pneumoniae* were selected for this investigation based on genetic propensity for attachment to an iron substrate (CS), potential for tolerance to harsh chemical treatment, synthesis of extracellular polymeric substances (EPS), occurrence in natural and industry settings (marine environments) and ability to thrive under marine-simulating laboratory conditions (determined experimentally, growth curve data not presented). Microbial attachment and its inhibition was evaluated in two different surface conditions: CS with pre-oxidised surface finish (covered with iron oxides) and CS with freshly ground surface finish.

## Methods

### Microbial isolates

Three strains were employed for this study; *Shewanella chilikensis* DC57*, Pseudomonas balearica* EC28 and a laboratory strain of *Klebsiella pneumoniae. S. chilikensis* DC57^26^ *and P. balearica* EC28^27^ were recently isolated from corroded steel in an industrial facility. Bacteria were grown in pure culture and harvested during log phase at between 48 and 96 h of incubation. The media for cell cultivation was an artificial seawater (ASW)^28^ with the addition of Bacto™ casamino acids (3 g/L w/v), sodium pyruvate (3 g/L w/v), D + glucose (3 g/L w/v) and ammonium nitrate (3 g/L w/v). Cell counts were performed manually using a Neubauer haemocytometer. Cells were then washed three times in ASW at 12,000 rpm. Each pellet was resuspended in ASW and allowed to incubate for one hour at 30 °C before direct introduction into reactor media. The final cell number used for all reactors was 10^6^ cells/mL of each isolate.

### Synthesis of CTA-4OHcinn

**Figure 1:**
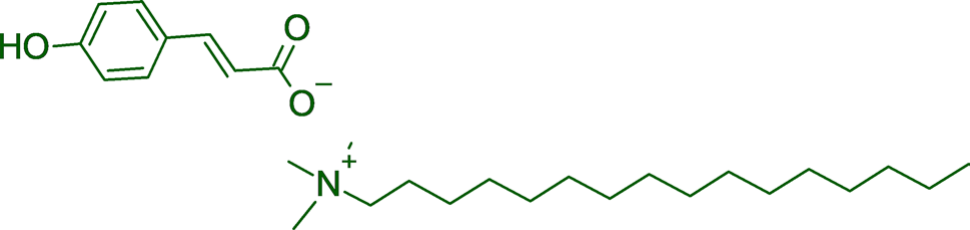
The molecular structure of CTA-4OHcinn used in this study.

A stirring solution of p-Coumaric acid (50 mmol, 8.2 g) in ethanol (75 mL) was slowly added to a solution of sodium hydroxide (50 mmol, 2 g) in ethanol (50 ml) at room temperature. After addition, the reaction was allowed to stir for two hours. The solution was then filtered and the product dried to collect the intermediate salt, sodium cinnamate. In the second step, sodium cinnamate (33.5 mmol, 6.23 g) was dissolved in an amount of water, then slowly added to a stirring solution of silver nitrite (33.5 mmol, 5.70 g) in water. After completion of the reaction, the solution was allowed to stir for a further hour in dark conditions (covered by aluminium foil). The product was filtered off and added to 50 mL of methanol. The provided solution was added slowly to a stirring solution of cetrimonium bromide (22.35 mmol, 8.15 g) in methanol (50 mml). The reaction was carried out at 80 °C in dark conditions for a week. Finally, AgBr and any unreacted silver cinnamate were firstly filtered off by filter paper and later by syringe filter. The methanol was evaporated through a rotary evaporator and later dried for 48 h in vacuo to get CTA-4OHcinn. The structure of the final product, CTA-4OHcinn is shown in Fig. 1. ^1^H NMR (400 MHz, CD_3_OD); δ 7.39 (d, J = 15.1 Hz, –CH=CH–, 1H), 7.40 (d, J = 9.5 Hz, ring, –HC=CH 2H), 6.8 (d, J = 8.63 Hz, ring, 2H), 6.34 (d, J = 16.82 Hz, HC = CH, 1H), 3.33 (m, NCH_2_, 2H), 3.11 (s, N(CH_3_)_3_, 9H), 1.77 (m, NCH_2_CH_2_, 2H), 1.30 (m, CH_2_, 26H), 0.91 (t, J = 6.57 Hz, CH_2_CH_3_, 3H) ppm. ^13^C NMR (100 MHz, CD_3_OD); δ 174 (COO), 158.6 (ring carbon attached to OH), 140.6 (ring C), 128.9 (ring C, C–C(OH)–C), 127.1 (C=CH–COO), 121.5 (C=C–COO), 115.2 (ring C), 66.49 (NCH_2_), 52.1 (N(CH_3_)_3_), 32 (CH_2_), 29.4 (CH_2_), 29.3 (CH_2_), 29.2 (CH_2_), 29.1 (CH_2_), 29.0 (CH_2_), 28.8 (CH_2_), 25.9 (CH_2_), 22.5 (CH_2_), 22.3 (CH_2_), 13.1 (CH_3_) ppm. ES^+^ m/z 285.3 ((CH_3_)_3_ N(C_16_H_33_)^+^, ES^−^ m/z 163.0 (*trans*-4-hydroxycinnmate)^−^. Anal. Calculated for C_28_H_50_N_1_O_3.5_; C, 73.64; H, 11.04: N, 3.06. Found: C, 73.74; H, 10.71: N, 2.66.

#### Material characterization

High Resolution Mass Spectra (HRMS) were obtained on an Agilent 1200 series HPLC system, and the sample of CTA-4OHcinn was sent to The Campbell Microanalytical Laboratory, New Zealand for elemental analysis. The results of this analysis can be viewed elsewhere^25^.

### Sample preparation and surface finish

**Figure 2:**
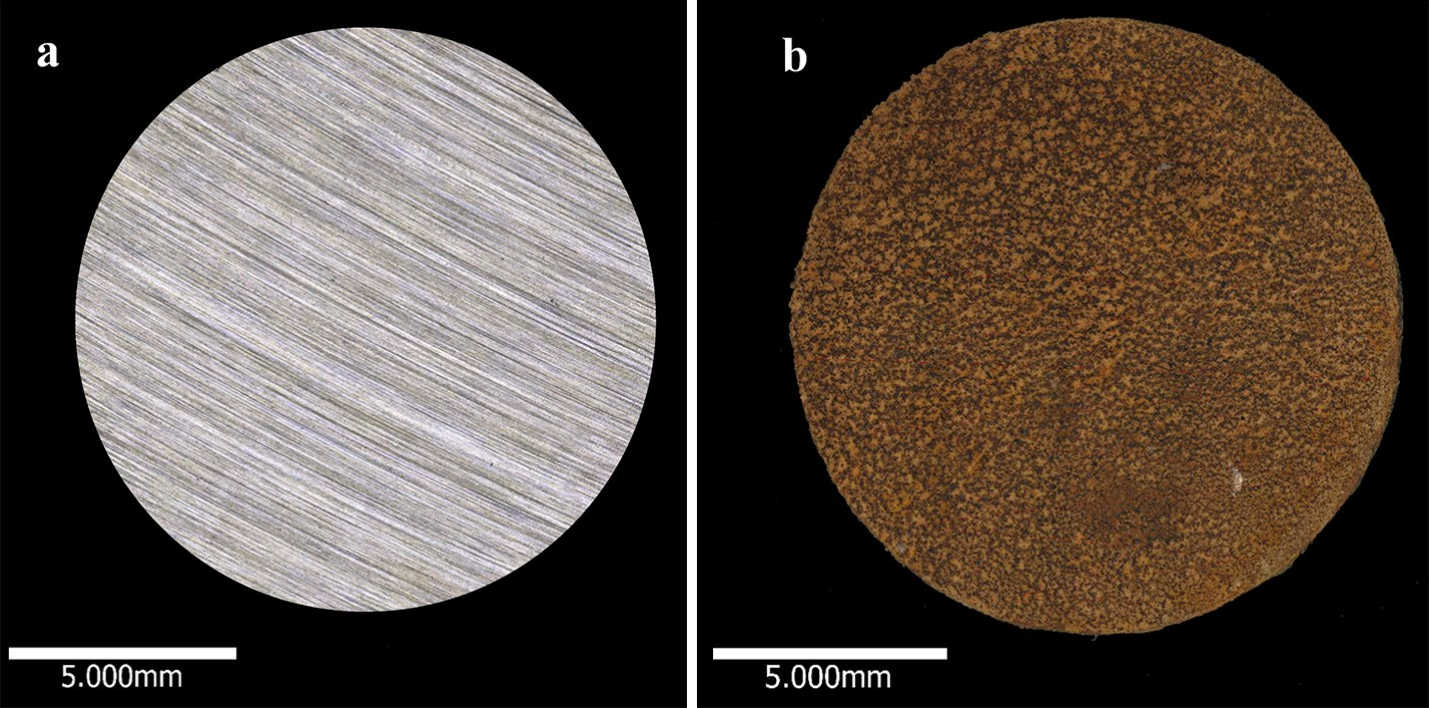
Micrographs of the entire wet-ground surface (**a**) and oxidised surface (**b**).

CS coupons (AISI 1030) of surface area 1.34 cm^2^ were wet-ground to 120 grit finish (SiC grit paper), washed in deionized water, degreased using absolute ethanol and dried under nitrogen gas. Coupons were UV irradiated for at least 10 min each side before fixing to a rod and transferring into a Centre for Disease Control (CDC) reactor. Coupons prepared by this method served directly as wet-ground surface finish coupons. These coupons were not visually corroded (Fig. 2a). To generate the oxidised surface finish, wet-ground coupons were transferred to a reactor with sterile ASW at 30 °C and agitated at 50 rpm for at least 12 h at atmospheric pressure of air to establish an iron oxide film. After oxidation had occurred, the reactor was then flushed with nitrogen for one hour (Fig. 2b). At this time, freshly wet-ground coupons were also introduced to the same reactor. This procedure (both surfaces evaluated using the same reactor) was performed to ensure the same population of cells was evaluated, to limit bias associated with cell counting and ensure no favourable attachment occurred as a result of the experimental design. Coupons representing oxidised surfaces were placed on opposite sides of the reactor to avoid influence of oxides on wet-ground surfaces. The oxidised and wet-ground surfaces represented the working surface for inhibitor evaluation.

### Experimental setup

Experiments were conducted using CDC reactors over a 24 h period spanning from inoculation of microorganisms to coupon sampling. Anaerobic conditions were generated by bubbling with pure nitrogen gas at atmospheric pressure. Deoxygenated ASW adapted from Eguchi et al.^28^ pH 7.4 was used as test solution for all experiments, with the only modification being the reduction in calcium chloride concentration from 1.5 to 0.1 g/L in order to limit precipitation. No additional supplements were added to the test solution. Passive attachment (settling) of molecules and bacterial cells was prevented by suspending the coupons vertically. The test solution was maintained under constant agitation at 50 rpm using an inbuilt magnetic stirrer and a temperature of 30 °C was generated using a hotplate. Fluid motion allowed non-motile bacteria (*K. pneumoniae*) access to the metal surfaces and ensured iron oxide layers were firmly associated with the surface before evaluating attachment. CTA-4OHcinn was added to the reactors 24 h before inoculation with the bacterial isolates to allow surface interaction before addition of microorganisms. CTA-4OHcinn was completely dissolved in ASW at 50 °C with agitation for 1 h. The solution was added to the reactors at a 10 mM concentration and gently agitated. The reactors were then inoculated with the bacterial isolates and incubated for 24 h after which time the coupons were removed and gently washed in phosphate buffered saline (PBS: Sigma, pH 7.4). Washed coupons were placed into 10 mL of PBS for CFU analysis.

### Confocal laser scanning microscopy (CLSM)

A Nikon A1+ confocal microscope was used for all CLSM analysis. Syto9™ and propidium iodide (PI) were purchased through Invitrogen™ as the Filmtracer™ LIVE/DEAD™ Biofilm Viability Kit. All experiments were conducted using a pinhole radius of 1.2 AU. Stains were mixed in phosphate buffered saline (PBS; pH 7.4) and coupons incubated in 200 μL of the stain mixture for 10 min before removing excess stain and inverting on a dish with central depression of radius 1 cm and covered by a glass cover slip (ibidi^®^, Germany). Controls were conducted to eliminate the possibility of auto-fluorescence (Supplementary Fig. 1). All micrographs were generated with a 20× objective for uniformity and to capture a large surface area of each coupon (600 µm^2^). Images were captured sequentially using a 489.3 nm laser and a 500–550 emission filter for Syto9™ and 561 nm laser with a 570–620 nm emission range for propidium iodide. Signal bleed-through between channels was reduced by acquiring z-stacks using separate tracks for emission and excitation paths. The area acquired also remained constant for all confocal microscopy.

Post-image analysis was conducted using IMARIS (Bitplane) software. The average ratio of live to dead cells and biofilm compactness was calculated for the oxidised surfaces using a representative micrograph from each microorganism before and after CTA-4OHcinn application, providing triplicate results for the surface evaluated. Post-image analysis was also performed on wet-ground surfaces before and after CTA-4OHcinn application (data not shown). The parameters evaluated here provide information on the disruption to cell density (compactness) and disruption to cell membranes associated with the compound application^29^.

### Scanning electron microscopy (SEM)

Coupons were lightly rinsed in pre-warmed PBS (30 °C, Sigma, pH 7.4) before fixing them in a 2.5% glutaraldehyde solution and dehydrating in an ethanol series as described elsewhere^30^. Coupons were then incubated for 22 h at 4 °C, removed and dried under nitrogen before coating with 9 nm of platinum. Coupons were micrographed using a Tescan MIRA-3 variable-pressure field emission scanning electron microscope at an emission voltage starting at 5 kV. *K. pneumoniae* attached to wet-ground surfaces were imaged at 8 kV to minimise charging.

### Cell quantification

Colony forming units (CFUs) were quantified for coupon surface area following existing standards^31^. Nutrient agar plates containing ASW supplemented with Bacto™ casamino acids (3 g/L w/v), glucose (3 g/L w/v), sodium pyruvate (3 g/L w/v) and ammonium nitrate (3 g/L w/v) were first evaluated for rapid growth of all isolates in aerobic conditions (countable colonies after 2 days). Coupons were removed from reactors and gently rinsed with PBS (Sigma, pH 7.4) before transferring to centrifuge tubes containing 10 mL fresh PBS. Cells were stripped from the surface by sonication as described elsewhere^30^. Plates were incubated at 30 °C for 2 days until visible colonies appeared. To confirm CFU/mL, most probable number (MPN) calculations were performed as described elsewhere^31^.

### Statistical analysis

A one-way ANOVA statistical analysis was conducted on triplicate CFU calculations using PAST (V4.83)^32^ to determine if microbial attachment rates to oxidised and wet ground surfaces was significantly different. Results returning a p-value ≤ 0.05 was considered significant.

### Oxidised surface analysis

Raman spectroscopy measurements to determine the oxide layer composition formed on oxidised carbon steel surfaces were performed using an Alpha300RA+ confocal Raman microscope (WITec GmbH, Ulm, Germany) equipped with a 20×/0.4 NA objective (Zeiss, Germany) and a an excitation laser with a wavelength of l = 532 nm. Single spectra were acquired at 50 ms integration times and averaged over 100 accumulations. Triplicate coupons were prepared as described previously (see Sect. “Sample preparation and surface finish”) with the exception of using a finer wet-ground finish for this analysis (600 grit).

## Results

### Effect of surface finish on bacterial attachment

**Figure 3:**
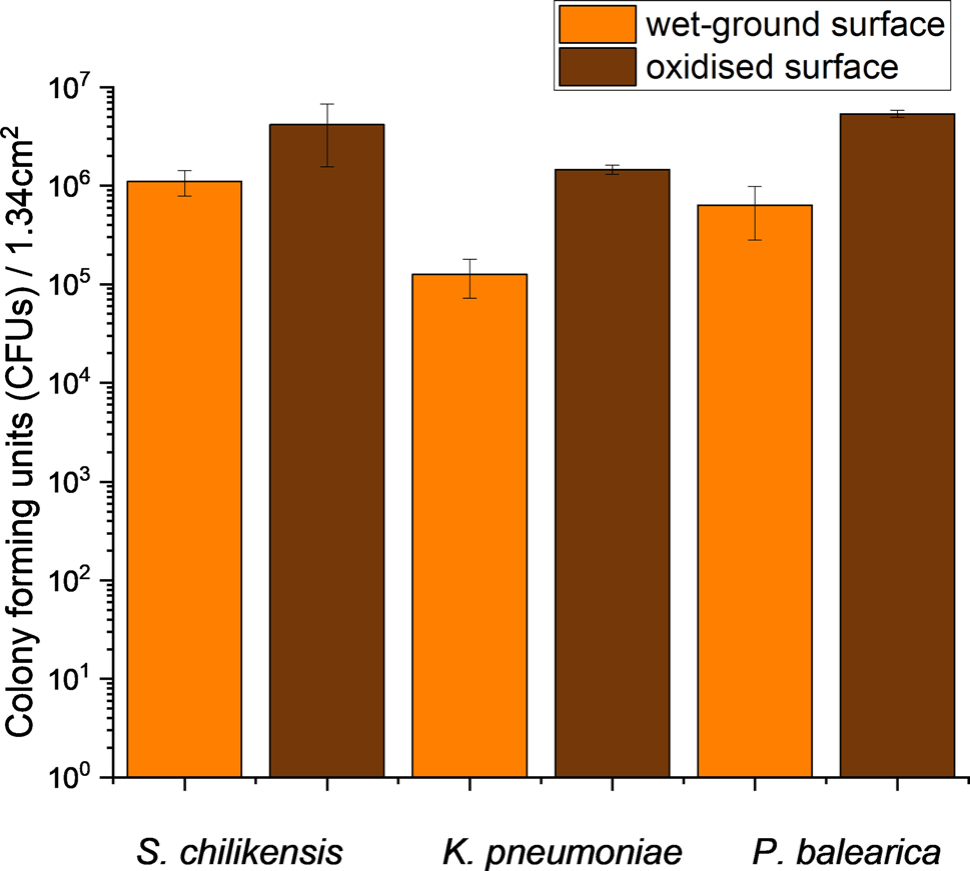
Direct cell quantification performed on oxidised surfaces and wet-ground surfaces after 24 h of exposure. Results are based on triplicate surface counts for each microorganism and surface type, and are expressed as CFUs per 1.34 cm^2^. Error bars represent the standard deviation of triplicates.

Oxidised and wet-ground surfaces were evaluated as substrates for attachment of the three bacterial strains. Results of quantification of sessile microorganisms as CFUs are presented in Fig. 3. All bacterial strains attached to oxidised surfaces in significantly higher numbers than wet-ground surfaces (p = 0.002). On average, over 4.16 × 10^6^ CFUs were measured for *S. chilikensis* on the oxidised surface, while 1.11 × 10^6^ CFU of *S. chilikensis* were obtained from the wet-ground surface. For *K. pneumoniae,* 1.46 × 10^6^ viable cells attached to oxidised surfaces as opposed to 1.26 × 10^5^ CFUs for the wet-ground surface. A similar trend was observed for *P. balearica* (5.36 × 10^6^ CFUs for wet ground surface compared with 6.33 × 10^5^ CFUs). Under conditions provided in the present work, oxidised surfaces were more favourable for bacterial attachment. However, the degree of difference between those surfaces as expressed in CFUs varied depending on the isolate. *S. chilikensis* viable cells attached just 3.7 times greater to oxidised surfaces than a wet-ground surface, while *K. pneumoniae* cells were 11.5 times more likely to attach to an oxidised surface. *P. balearica* cells exhibited an 8.5 times greater affinity to the oxidised surface. Lastly, *P. balearica* and *S. chilikensis* attached to both surfaces in greater numbers than *K. pneumoniae*.

**Figure 4:**
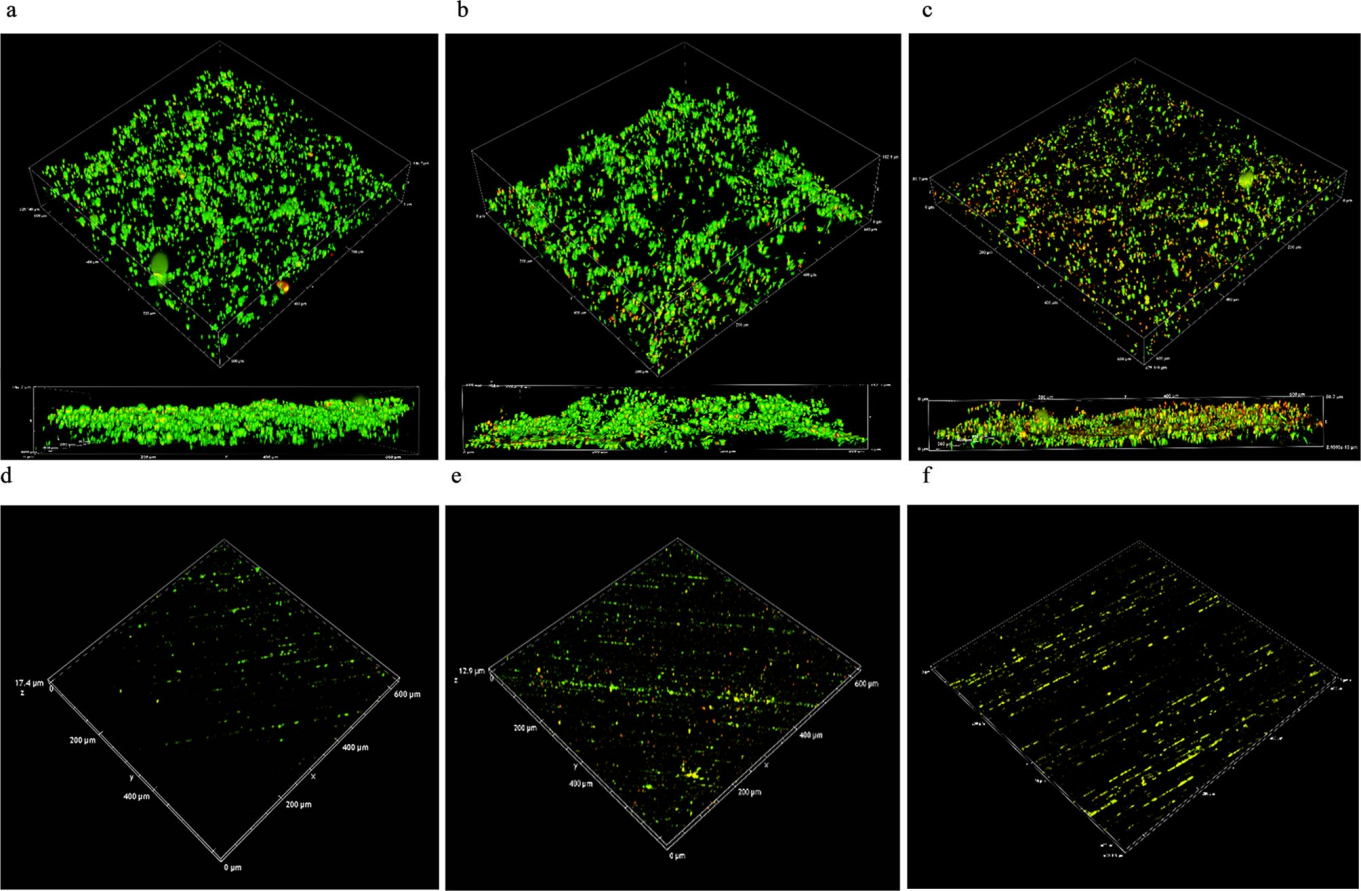
LIVE/DEAD confocal micrographs of attached cells on oxidised surfaces compared to wet-ground surfaces using a ×20 objective. Micrographs (**a**–**c**) represent *S. chilikensis*, *K. pneumoniae* and *P. balearica* attached to oxidised surfaces respectively. Micrographs (**d**–**f**) represent the isolates attached to wet-ground surfaces.

CLSM generated a series of z-stacks for each surface, representing a surface area of 600 µm^2^. Figure 4a–c shows oxidised surfaces and attached *S. chilikensis, K. pneumoniae* and *P. balearica* cells, respectively. The technique was also applied to wet-ground surfaces seen in Fig. 4d–f. CLSM confirmed trends observed in CFU calculations; the bacteria evaluated in this study are more likely to attach to oxidised CS compared to wet-ground CS surfaces in seawater solution.

**Figure 5:**
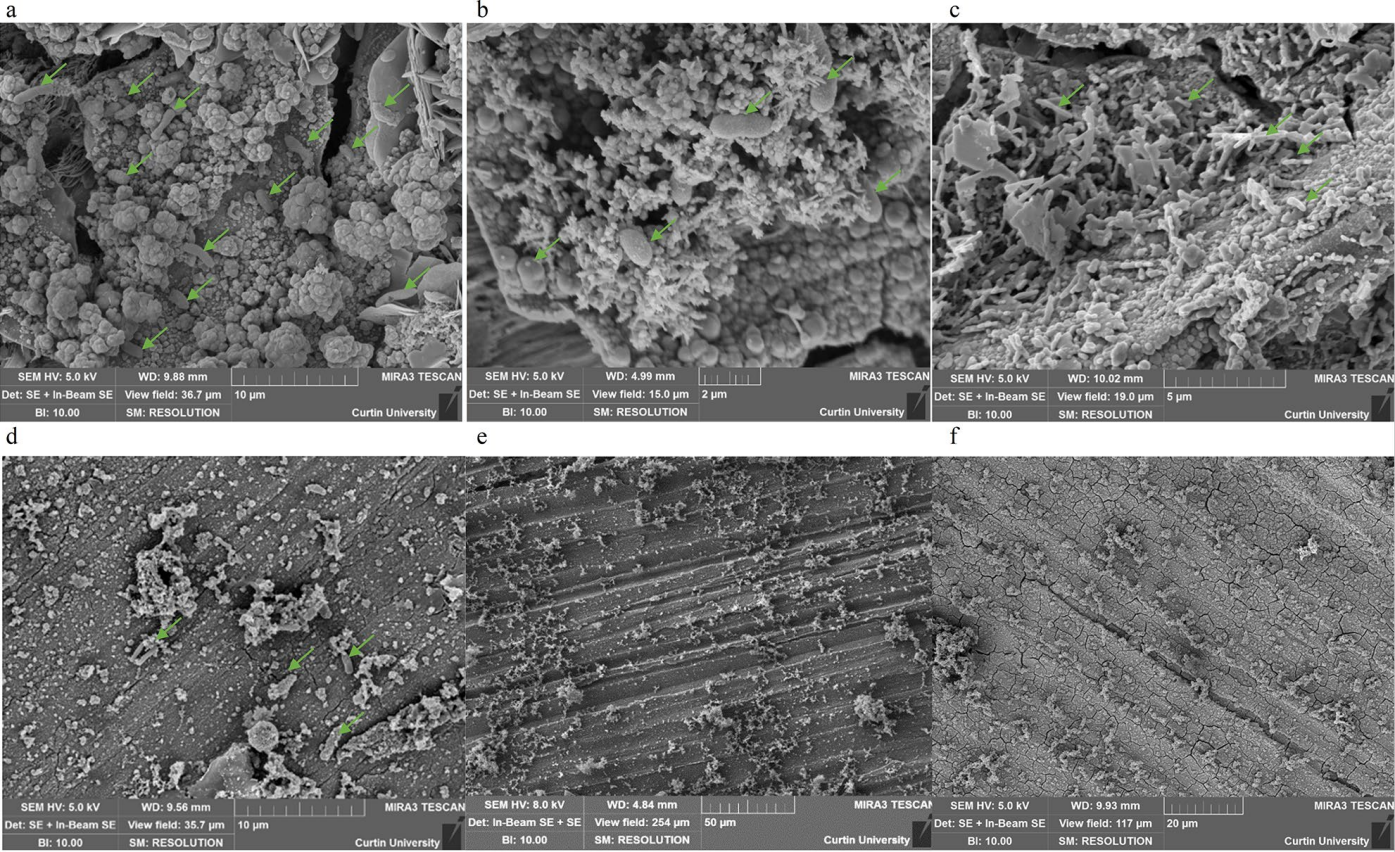
Representative SEM micrographs of oxidised and wet-ground control surfaces. Arrows indicate structures resembling bacterial cells. Micrographs (**a**–**c**) represent *S. chilikensis*, *K. pneumoniae* and *P. balearica* attached to oxidised surfaces respectively. Micrographs (**d**–**f**) represent the isolates attached to wet-ground surfaces.

SEM was conducted to directly observe bacterial attachment to wet-ground and oxidised surfaces. Figure 5a–c shows oxidised surfaces exposed to *S. chilikensis, K. pneumoniae* and *P. balearica,* respectively. Micrographs in Fig. 5d–f represent attachment of these strains to wet-ground surfaces. The micrographs clearly demonstrate the heterogeneous surface of the oxidised coupons, which corresponds to greater bacterial attachment. Conversely, the wet-ground surfaces hosted smoother topography with less bacterial attachment. In agreement with previous results, SEM results indicate that bacteria preferentially attached to the oxidised surface over the wet-ground surface.

### Effect of CTA-4OHcinn on bacterial attachment

Cell quantification, CLSM and SEM were employed to evaluate the effect of the inhibitor on the attachment of the isolates to oxidised and wet-ground surfaces. For all inhibitor analysis, a concentration of 10 mM of CTA-4OHcinn was evaluated. Results of quantification of the isolates attached to oxidised and wet-ground surfaces in the presence of 10 mM of CTA-4OHcinn are provided in Table 1. CFU quantification was represented by the mean count of three replicate surfaces. In the presence of CTA-4OHcinn, bacteria experienced a significant reduction in cell attachment to the oxidised surface, i.e., 100% reduction for *S. chilikensis*, 99.7% for *K. pneumoniae* and 99.8% for *P. balearica*. The rates of cell attachment on wet-ground surface were reduced by 100%, 96.6% and 98.9% respectively (See Supplementary Fig. 2). Although similar reductions to attachment were observed between the two surface types, oxidised surfaces had initially higher numbers of attached cells. Although oxidised and wet-ground surfaces demonstrated marked difference in attached cells before biocide exposure, attached *K. pneumonia* and *P. balearica* to these surfaces were reduced after exposure to CTA-4OHcinn to < 1.17 × 10^4^ CFUs per 1.34 cm^2^. Therefore, an initially larger quantity of attached cells did not necessary result in higher viable cell number after CTA-4OHcinn exposure.

**Table 1:** Cell numbers attached to oxidised and wet-ground surfaces before and after exposure to 10 mM of CTA-4OHcinn for each isolate.

	Isolate	Without CTA4OHcinn	With CTA-4OHcinn	% reduction
Oxidised surface	*S. chilikensis*	4.17 × 10^6^	0.00	100
	*K. pneumoniae*	1.46 × 10^6^	4.33 × 10^3^	99.7
	*P. balearica*	5.37 × 10^6^	1.17 × 10^4^	99.8
Wet-ground surface	*S. chilikensis*	1.11 × 10^6^	0.00	100
	*K. pneumoniae*	1.27 × 10^5^	4.33 × 10^3^	96.6
	*P. balearica*	6.33 × 10^5^	6.67 × 10^3^	99.0

Original values for surface colonisation are taken from Fig. 3.

**Figure 6:**
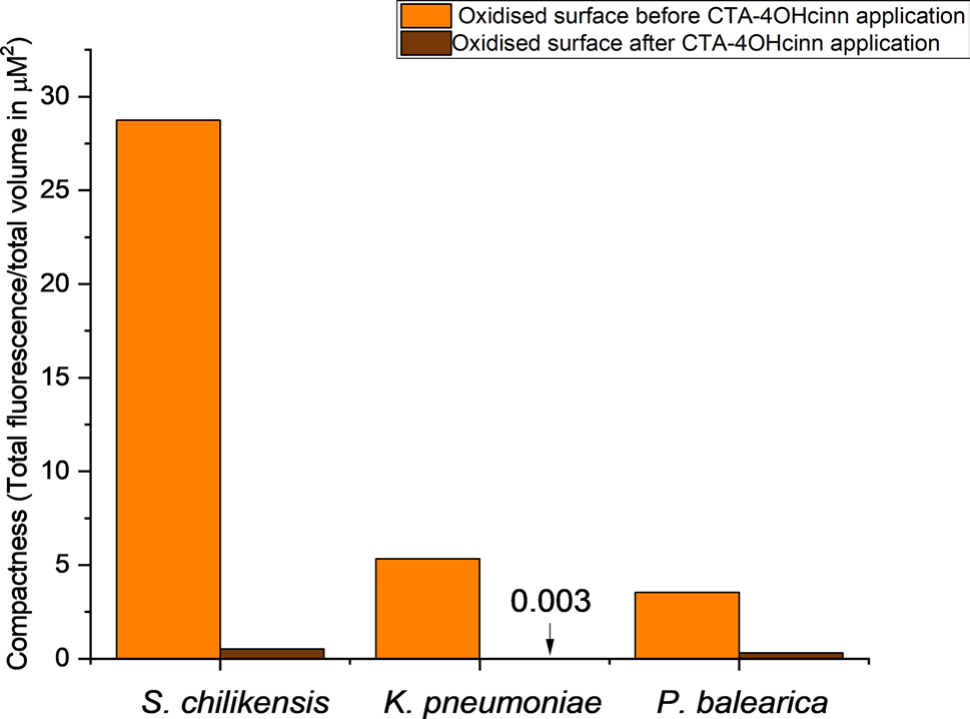
Biofilm compactness of oxidised surfaces expressed as total fluorescence/total volume in µM^3^ of substrate surface area.

Reduced cell numbers in the presence of 10 mM CTA-4OHcinn were supported by CLSM observations and post-image analysis (Fig. 6). Although live/dead cell ratios remained similar, especially for *P. balearica* (Table 2), compactness of all biofilms (cell density) was strongly impacted by the application of the compound. Reductions to compactness values were 56 x, 18,000× and 11× for *S. chilikensis, K. pneumoniae* and *P. balearica* respectively. In micrograph 3D reconstructions, decreased SYTO9™ (*live* signal) was observed for all strains and surface finishes, returning signal intensity to levels similar to abiotic controls (See Supplementary Fig. 1). Increased PI (*dead* signal) was also observed, especially for *P. balearica* cells on oxidised surfaces. All results indicate a reduction in cell attachment, cell membrane integrity and cell viability, regardless of the isolate or surface finish.

**Table 2:** Live and dead cell fluorescence intensity with live and dead cell ratio for oxidised surfaces before and after application of CTA-4OHcinn.

	*S. chilikensis*		*K. pneumoniae*		*P. balearica*	
	Before	After	Before	After	Before	After
Live cells	82%	66%	88%	55%	75%	76%
Dead cells	18%	34%	12%	45%	15%	14%
Live cell ratio	41:50F	33:50	22:25	11:20	3:4	19:25

SEM microscopy supported the data generated by CLSM and cell quantification. On all wet-ground surfaces, SEM analysis indicated total inhibition of bacterial attachment after biocidal application (Supplementary Fig. 3). *S. chilikensis* on wet-ground surfaces exposed to test solution without CTA-4OHcinn (see Fig. 5d), had low numbers of attached cells, whereas no cell attachment was observed on wet-ground surfaces for the other isolates (representative micrographs *e* and *f*) even though CFUs were extracted from those samples. While SEM images are localised by nature, the images shown represented the surface and highlight the limited cell attachment on the wet ground samples. Conversely, oxidised surfaces were associated with aggregation of cells attached to the oxides (see Fig. 5a–c). Upon exposure to CTA-4OHcinn, very few *P. balearica* cells were observed on the oxidised surface (Supplementary Fig. 3f). This is in agreement with confocal micrographs (Supplementary Fig. 2c), highlighting the survival of some *P. balearica* cells in the presence of CTA-4OHcinn.

The results from CFU counting, CLSM and SEM indicate that CTA-4OHcinn was effective at reducing bacterial viability and cell attachment on both wet-ground and oxidised surfaces of carbon steel.

#### Surface oxide analysis

Spectra obtained from random locations across three coupons contained peaks at wavelengths 212, 273 and 384 rel. 1/cm (Supplementary Fig. 4), likely corresponding to bands produced by goethite and hematite^33,34^.

## Discussion

Increased environmental awareness and regulation stringency has forced marine industrial processes, particularly oil and gas operations, to switch to greener biocide alternatives. In a recent study by Ghorbani et al.^25^, the authors described the novel OCI designated CTA-4OHcinn (Fig. 1) a compound expected to act both as a biocide and corrosion inhibitor simultaneously. The compound demonstrated corrosion inhibition efficiencies on CS (AISI 1030) when applied at 5, 7.5 and 10 mM of 95%, 95%, and 96% after 30 min^25^. The effective inhibition capacity is predicted to be the result of *trans*-4-hydroxy cinnamate or cetrimonium ions, or both, interacting with the surface and suppressing anodic cell development^25^. Specifically, through NMR diffusion studies and electrochemical methods the authors predicted cylindrical micelle formation of the compounds on the fluid/CS interface, leading to bilayer film formation as the mechanism of corrosion inhibition. Since optimal corrosion prevention was previously observed at a concentration of 10 mM, this concentration was evaluated for antimicrobial capacity in the present study.

Along with CI capacity, film forming OCIs such as CTA-4OHcinn may also function as an effective biocide. CTA contains a quaternary ammonium functional group (QAC), which have been frequently employed as disinfectants in the health sector for decades^35^. Major reductions in cell viability and attachment is expected after exposure to CTA compounds, supported by previous reports on organic film-forming QACs^35,36^.

On the contrary, it has also been suggested that utilising QACs on metal surfaces can promote bacterial attachment. Work by Mousavi et al.^37^ suggests increased attachment rates observed in *Pseudomonas putida* to steel surfaces coated with QACs was the result of alteration of the surface microstructure to a more porous texture, providing a viable landscape for attachment of the isolate. Acquisition of tolerance mechanisms to QACs is also of increasing concern, which may lead to enhanced attachment^37^. QACs still present low LD_50_ values compared with many other available alternatives (e.g., glutaraldehyde)^21^ and are therefore safer and more desirable from an environmental and holistic approach if effectiveness is enhanced. In a recent study, LD_50_ values of CTA-4OHcinn in human keratinocyte (skin) and duodenum (intestinal) cell lines were compared against cetrimonium bromide (CTA-Br), a well-established and safe additive to disinfectants and cosmetics^38^. Results of this study conclude lower cytotoxicity in CTA-4OHcinn-exposed cell lines compared with CTA-Br, as well as a lower absorption rate into the cell layer^38^. QACs such as CTA-4OHcinn with biocide-enhancing additives therefore represent promising MIC mitigating compounds moving into a more environmentally sensible future.

In addition to lower relative toxicity compared to similar compounds and other biocide classes, the incorporation of certain compounds has been demonstrated to act synergistically to enhance the biocidal effectiveness of QACs, or introduce new functions. Seter et al. described the OCI cetrimonium nalidixate, with corrosion inhibition properties, which could also limit biofilm formation at the interface of carbon steel^21^. In the present study, we assessed the novel OCI CTA-4OHcinn consisting of a cetrimonium cation paired to an organic carboxylic acid, 4-OH cinnamate. The incorporation of 4-OH cinnamate to the QAC is hypothesised to enhance its biocidal capacity, adding a function normally requiring separate treatment. Although ongoing, it is speculated that bacterial attachment is limited by a synergistic action; that is, the cetrimonium cation provides rapid surface protection bringing 4OHcin with it, where it can disrupt cell membrane integrity^39^. In the present work, the increased PI signal in confocal micrograph post-image analysis after compound application supports this mechanism.

To evaluate biocidal capacity, studies still frequently include the calculation of minimum inhibitory concentration on planktonic cells, despite the well-established difference between planktonic MIC and minimum biofilm elimination concentrations (MBEC)^40^. Additionally, the vast majority of microorganisms are found living at an interface in biofilms^12^. In this phenotype (unlike planktonic lifestyles) microorganisms can begin to initiate MIC or biofouling. It is therefore necessary to evaluate the efficiency of biocides to prevent attachment, adhesion and biofilm maturation of native bacteria to the metal surface. Additionally, attachment and adhesion of bacteria may be highly variable in marine conditions owing to the diversity of attachment phenotypes, surface properties and fluid content, among other factors^41^. In the present study, bacterial attachment was selected for evaluation of biocidal activity since both biofilm formation^15^ and MIC^42^ rely on initial colonisation of the surface.

Surface oxidation was considered an important parameter when evaluating attachment and biocide activity. It is now well established for example that bacteria gain a range of metabolic benefits from the presence of surface ions such as FeII^+^ and FeIII^+^^43^, usually present as iron oxides on CS. When present in the environment (such as pipeline fluids), bacteria may be initially attracted to a metal surface^44^, a phenomena evaluated in the present work using a pristine CS control compared against a pre-corroded CS surface. Based on the metabolic benefits afforded by FeII^+^ and FeIII^+^ and enhanced surface roughness, it is hypothesised that bacteria preferentially adhere to oxidised surfaces, and that CTA-4OHcinn will impede bacterial attachment to both surfaces.

Hence, the secondary aim of this study was to underpin any differences in bacterial attachment to these surfaces in the presence and absence of the CTA-4OHcinn. The context of this study is marine infrastructure where both MIC and biofouling are prevalent concerns. Therefore three native marine bacteria were selected to evaluate the compound. These isolates were selected based on the following considerations; (a) presence or expression of genes that may promote adhesion to CS, (b) exhibiting diversity in morphology, (c) proliferate under marine-simulating conditions in a reasonable timeframe and (d) express facultative respiration. Lastly, the authors recognise that MIC is a complex problem that may be driven by a variety of bacterial species^8^. The selection of strains employed in this study therefore attempts to capture genetic and phenotypic differences in the selected strains.

EPS, especially viscous mucous expressed by members of the *Klebsiella* genus assist in attachment, chemical (antimicrobial treatment) protection and tolerance to environmental stressors such as heat, host cytokine responses and desiccation. *K. pneumoniae* was selected for this study for its ability to thrive in marine conditions, metabolic traits and diverse range of virulence factors; especially a thick mucus capsule, which were expected to provide inhibitor protection and enhanced attachment over less prolific EPS producers.

*Shewanella* are known for their strong affiliation with metallic surfaces. *S. odeniensis* for example is attracted to iron oxide surfaces where it expresses proteins such as cytochromes to reduce FeIII^+^. *Shewanella chilikensis* strain DC57 was isolated from a floating production storage and offloading unit (FPSO) where it was thought to be involved in MIC^26,45^. Very little has been revealed about *S chilikensis* since its recent discovery by Sucharita et al. in the Chilika lagoon, India^46^. Work by Sucharita et al. ^46^ at the time of original isolation demonstrated tolerance of NaCl up to 8%, and based on its recent isolation from corroded industry equipment exposed to seawater (FPSO), *S. chilikensis* DC57 was expected to thrive in marine simulating conditions used in this study. *S. chilikensis* is a facultative anaerobe. Metabolic traits and genes associated with metal reduction present in the genome of this isolate, native location and previous findings of the isolate^26,45^, were selective criteria resulting in use of *S. chilikensis* DC57 for this study. The attachment and inhibition of this isolate on metallic substrates have not been investigated so far.

P*seudomonas* are frequently seen in the marine environment and nosocomial infections, especially model biofilm forming strain *P. aeruginosa* which has been described as one of the most clinically significant opportunistic pathogens^47,48^. *Pseudomonas* spp. can have a diverse range of metabolisms^49^ and virulence factors including mixtures of secreted biosurfactants^48^ providing them renowned chemical treatment tolerance. Studies have also revealed that the presence of metallic ions may also facilitate metabolic functions in some *Pseudomonas*^50^. Like *S. chilikensis*, limited research evaluates *P. balearica* despite being proposed as a new species 25 years ago^51^. The *Pseudomonas* strain used in this work, *P. balearica* EC28, was also isolated from an FPSO and expected to contribute to MIC^27^. This facultative anaerobic isolate can tolerate up to 8.5% NaCl and is considered a true marine isolate^49^, meeting the criteria for isolate selection in this study.

The primary aim of the present study was to assess the biocidal capacity of the new OCI CTA-4OHcinn. In this study attachment to pre-oxidised and wet-ground CS surfaces was evaluated for these three isolates in marine simulating conditions, providing control data for the evaluation of CTA-4OHcinn. Attachment rates were compared after a 24 h timeframe with and without exposure to 10 mM CTA-4OHcinn. This timeframe was selected to; (a) demonstrate the rapid capacity of marine bacteria to colonise metal surfaces, (b) distinguish the immediate differences in attachment tendency of the isolates to the different surfaces and (c) investigate the capacity of CTA-4OHcinn to prevent rapid attachment to these surfaces. Quantifying and visualising attached cells directly through a range of techniques (including CFU quantification, SEM and CLSM) provides a more detailed representation of biofilm forming units as opposed to planktonic cells which do not represent the more recalcitrant sessile counterparts likely to cause corrosion. Membrane integrity can be used as a measure of cell viability, however the tendency of PI to enter cells with compromised membranes can represent bias (for example, by entering the cytoplasm of viable cells undergoing membrane repair^52^). Therefore attachment was evaluated under constant agitation to ensure cell attachment was an active process, with cells damaged only due to action of the inhibitor.

Results indicate that attachment of marine bacteria to CS surfaces is effectively controlled by CTA-4OHcinn. Confocal micrographs of the isolates that had not been exposed to the compound showed a strong SYTO9™ (live) signal (82% average signal contribution) with PI (dead) contributing an average of 18% of the total signal. Some cell death is expected during initial colonisation of a surface, especially in *Pseudomonas* strains. Early attachment of *Pseudomonas* is likely to incorporate active cell lysis, a process responsible for excretion of compounds that promote attachment including extracellular DNA (exDNA)^53^. In the presence of CTA-4OHcinn, the signal from SYTO9™ was reduced to an average of 66%. For the *Shewanella* and *Klebsiella* strains on both surfaces, significant reductions in compactness and live:dead cell ratio were observed. The third isolate, *P. balearica*, indicated a 98% reduction in compactness with no significant change to the live:dead ratio in remaining cells. The signal from both stains was minimal after exposure, suggesting most non-viable or membrane-compromised bacteria did not remain attached to the surfaces. Although live and dead cell ratio was not heavily affected by the application of CTA-4OHcinn, compactness values demonstrated that the reservoir of viable cells on the surface is significantly reduced. A sharp reduction in compactness is in accordance with expectations, since the media was under constant agitation and cells not actively attaching to the surface would be removed by the stirring effect. In *Pseudomonas*, initial attachment to oxidised CS was also relatively uniform. A higher signal with PI was recorded in *Pseudomonas* on oxidised surfaces compared to other strains, supporting evidence that *Pseudomonas* strains actively lyse cells or secrete exDNA to condition a surface. DNA excreted during early attachment of *Pseudomonas* strains has a known affinity to iron oxides^54^.

Bacteria with membranes damaged by CTA-4OHcinn that are still viable may also be generated from the PI stain, adhered irreversibly before exposure to the OCI. CFU results expressing the reduction of cells after dosing (Fig. 3*)* confirmed that attachment of viable cells was controlled effectively by presence of 10 mM CTA-4OHcinn. Results from SEM further support the hypothesis that regardless of the isolate or surface employed, bacterial attachment is effectively controlled. However, bacterial attachment was not completely prevented by application of CTA-4OHcinn.

The study also hypothesised that bacteria prefer to adhere to pre-oxidised CS compared to wet-ground CS. Unsurprisingly when comparing oxidised and wet-ground surfaces in this study, all isolates overwhelmingly favoured attachment to the oxidised surface. Oxidised surfaces also demonstrated a greater biofilm compactness compared to wet-ground surfaces (Fig. 6a–c, Supplementary Fig. 2a–c). To better characterise this experimental surface, prepared oxidised coupons were analysed using confocal Raman spectroscopy. Peaks likely to correspond with common oxides haematite and goethite were observed (see Supplementary Fig. 4)^33,34^, however, due to high background fluorescence an intra-red laser is suggested for further work to determine the exact nature of the sample.

There are many factors that may influence attachment of individual strains to a surface, a topic which has been subject to extensive research. A review of such factors is provided by Goulter et al., including surface roughness and hydrophobicity^55^. Surface roughness of the oxidised surfaces employed for the present study can be seen in Fig. 2b. Typical surface structures deviated between − 5 and 65 µm from the reference point in oxidised surfaces compared to the freshly wet-ground surface, which deviated between − 6 and 6 µm. Additionally metal ions FeII^+^ and FeIII^+^, probably present on the pre-oxidised surface as iron oxides hematite and magnetite, provide metabolic advantages to many marine isolates. In the present work, a source of organic carbon was not provided in the test solution. FeII^+^ and FeIII^+^ could have promoted selective attachment based on an ability for chemolithotrophic metabolism, a well-established phenomenon in *Shewanella* strains^18^. In *Klebsiella* and *Pseudomonas*, research has demonstrated that addition of iron to cultures results in faster replication and formation of more robust biofilms^56,57^.

Direct cell quantification of wet-ground and oxidised surfaces was conducted to confirm viability observed in confocal microscopy and attachment observed through SEM. Numbers of viable cells initially attached to surfaces (in the absence of CTA-4OHcinn) confirmed that all isolates preferentially attached to oxidised surfaces (Fig. 3). For all strains, attachment to oxidised surfaces was present in numbers from 10^6^ to 10^7^ CFUs/1.34 cm^2^, demonstrating a large number of viable cells were present on the surface. Wet-ground surfaces hosted lower cell numbers than oxidised surfaces (10^5^ and 10^6^ cells/1.34 cm^2^). This was in accordance with confocal microscopy. SEM was conducted to directly examine the surface and the associations of cells with the distinctive surfaces, confirming increased attachment of the isolates to oxidised surfaces (Fig. 5).

## Conclusion

Bacterial attachment to carbon steels is a pervasive problem to oil and gas operations, resulting in biofouling and microbiologically influenced corrosion (MIC). To effectively control bacterial attachment, environmentally hazardous biocides are currently still employed in marine environments. As these compounds face international embargos, there is a drive to develop more sustainable and environmentally sensible biocide alternatives. This study introduces the novel, dual-action organic corrosion inhibitor (OCI) cetrimonium trans-4OH cinnamide (CTA-4OHcinn), which was evaluated for biocidal capacity against three bacterial isolates native to marine environments and previously implicated in MIC. Attachment of these isolates to 1030 carbon steel wet-ground and pre-corroded substrates was also evaluated and compared to determine the impact of iron oxide films on early bacterial colonisation. All isolates demonstrated preferential and enhanced attachment to carbon steel with an oxidised surface finish compared to the freshly wet-ground surfaces under the experimental conditions described. Results of direct viable cell quantification (CFU quantification), confocal microscopy, post-image analysis and scanning electron microscopy indicated that attachment of bacteria to these surfaces can be effectively controlled by the new OCI CTA-4OHcinn. The compound was applied at a 10 mM concentration, which was found to be optimal for corrosion inhibition in previous work. Based on these dual functions, this study provides the first evidence that CTA-4OHcinn has potential for use as a multi-functional OCI, and that biocidal function is not impacted by initial viable bacterial numbers and biofilm compactness on pre-oxidised carbon steel surfaces.

## Supplementary Information

Supplementary Information.

## Data Availability

The data supporting this study is available from the corresponding author under request.
