# Supplementary material for: Evaluation of a novel, multi-functional inhibitor compound for prevention of biofilm formation on carbon steel in marine environments
Source: Sci Rep. 2021 Aug 3;11:15697. doi: 10.1038/s41598-021-94827-9 (PMC8333064; doi:10.1038/s41598-021-94827-9)
Supplement: Supplementary file 1 — Supplementary Information. [file 41598_2021_94827_MOESM1_ESM.docx]

Evaluation of a novel, multi-functional inhibitor compound for prevention of biofilm formation on carbon steel in marine environments

Benjamin Tuck^a^, Elizabeth Watkin^b^, Maria Forsyth^c^, Anthony Somers^c^, Mahdi Ghorbani^c^, Laura L. Machuca^a^*

^a^Curtin Corrosion Centre, WA School of Mines: Minerals, Energy and Chemical Engineering, Curtin University, Kent Street, Bentley, WA 6102, Australia

^b^Curtin Medical School, Curtin University, Kent Street, Bentley, WA 6102, Australia

^c^Institute for Frontier Materials, Deakin University, Geelong, VIC, 3217, Australia

*Corresponding author: Laura L. Machuca; [L.Machuca2@curtin.edu.au](mailto:L.Machuca2@curtin.edu.au)

Confocal laser scanning microscopy controls:

To ensure Live/Dead micrographs (Syto9™ and PI) indicated cell viability, coupons were prepared and placed in a reactor as described in section 2 (Methods) without microorganisms. Background auto-fluorescence was determined on coupons stained with Syto9™ and PI through the same procedure (2.5 Confocal laser scanning microscopy). At least 10 micrographs were captured across the surface using a 20 x objective. Syto9™ and PI fluorescence was similar to surfaces micrographed after biocide application in *S. chilikensis* and *K. pneumoniae*, which was supported by CLSM observations (see *Sup. Figure 1*).

Wet-ground surfaces were also micrographed before and after application of CTA-4OHcinn (*Supplementary Figure 2 and 3*). Sup. Figure 2 shows confocal microscopic analysis of wet-ground surfaces with each isolate compared with polished coupons exposed to CTA-4OHcinn. A reduction in Syto9™ fluorescence is observed across all samples. In some samples (*Sup. Figure 2, e, f*) an enhanced PI (damaged cell) signal was observed.

Scanning electron microscopy:

SEM was performed on samples to visually identify cells adhered to the surface as well as the surface topology of coupons before and after exposure to CTA-4OHcinn. Results from experiments before exposure to the biocide can be seen in *Sup. Figure 3.* These results demonstrated an enhanced affinity of all three isolates to the oxidised surface, and low levels of attachment to wet-ground surfaces. Results from after exposure to the biocide presented fewer adhered cells to all surfaces, data in *Sup. Figure 3*. Wet-ground surfaces (*a, b and c*) appeared uncorroded and no live cells were observed. No live cells were observed on oxidised surfaces *d* and *e* (*S. chilikensis* and *K. pneumoniae*), and < 3 cells were observed across the working oxidised *P. balearica* surface after exposure to CTA-4OHcinn.

| 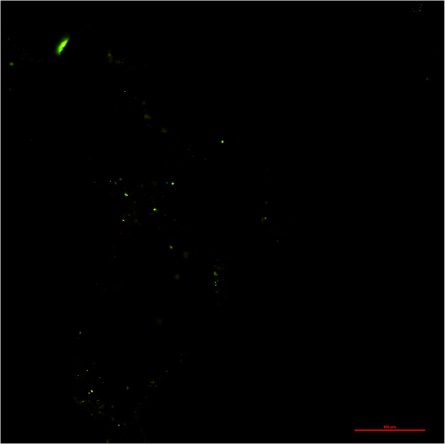a) location 1 | 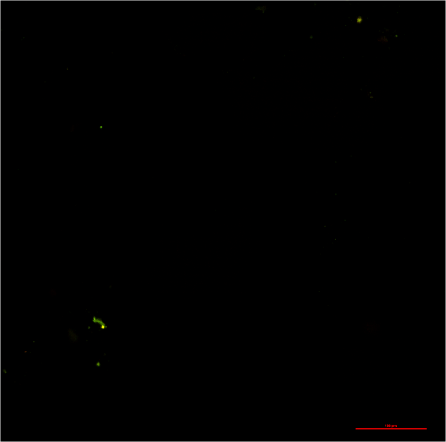b) location 2 | 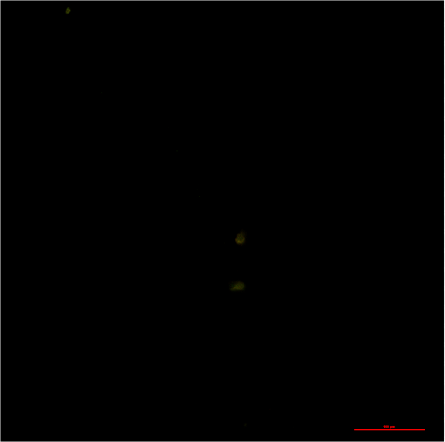c) location 3 |
| --- | --- | --- |

***Supplementary Figure 1:*** *Representative micrographs captured using a 20 x objective of background fluorescence levels after application of Styo9™ and propidium iodide to corroded CS coupons.*

| *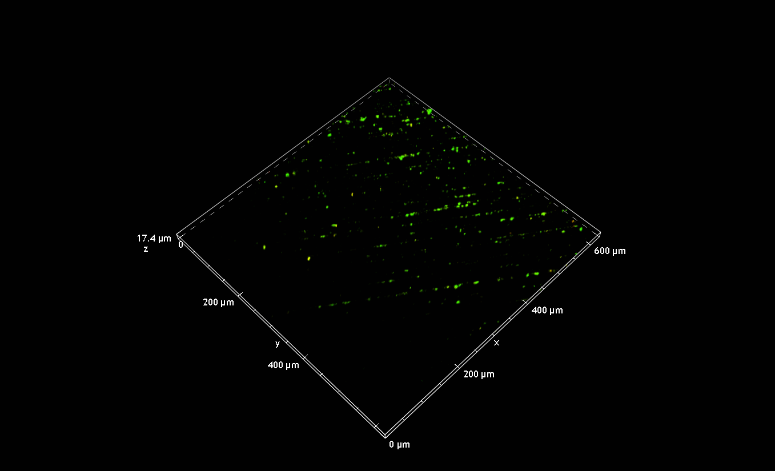a) S. chilikensis on wet-ground surface before biocide* | *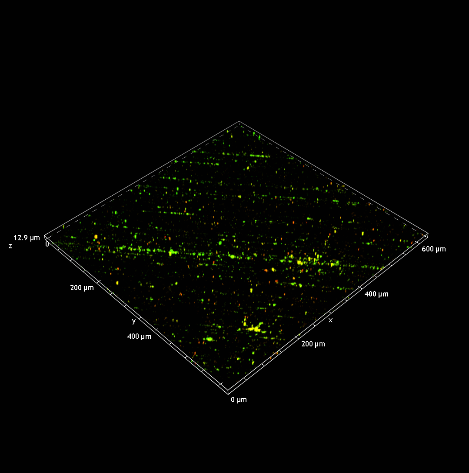b) K. pneumoniae on wet-ground surface before biocide* | *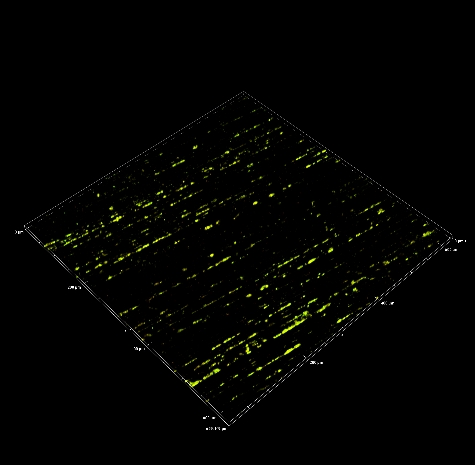c) P. balearica on wet-ground surface before biocide* |
| --- | --- | --- |
| *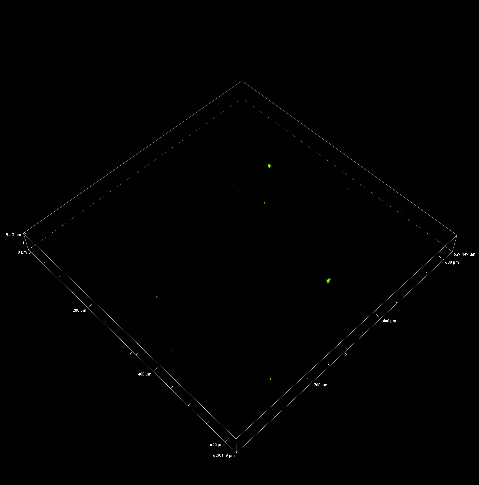d) S. chilikensis on wet-ground surface after biocide application* | *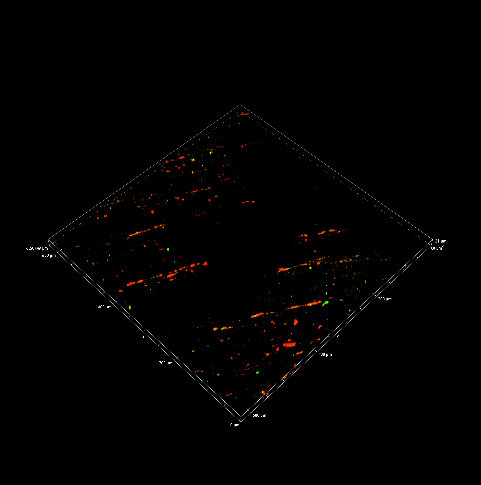e) K. pneumoniae on wet-ground surface after biocide application* | *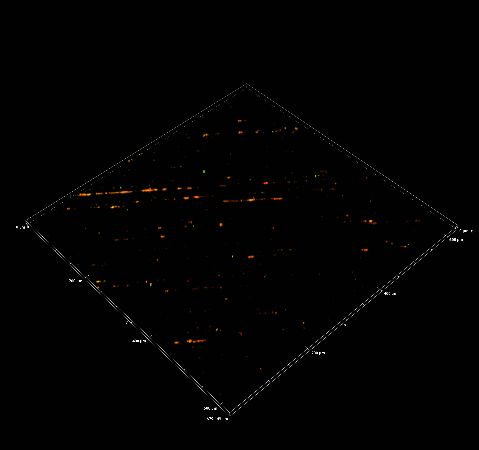f) P. balearica on wet-ground surface after biocide application* |

***Supplementary Figure 2:*** *Representative confocal micrographs of wet-ground CS surfaces before and after exposure to CTA-4OHcinn.*

| *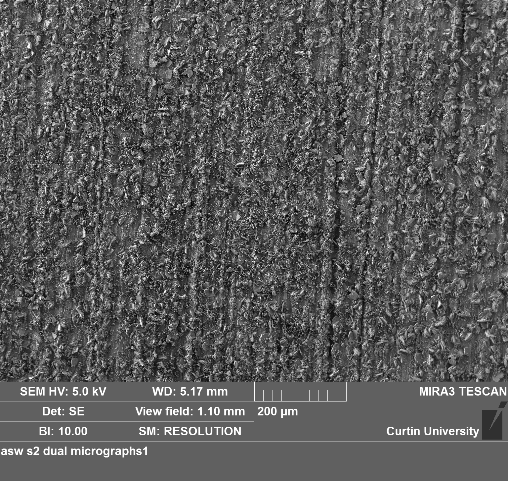a) S. chilikensis* on wet-ground surface after biocide application | *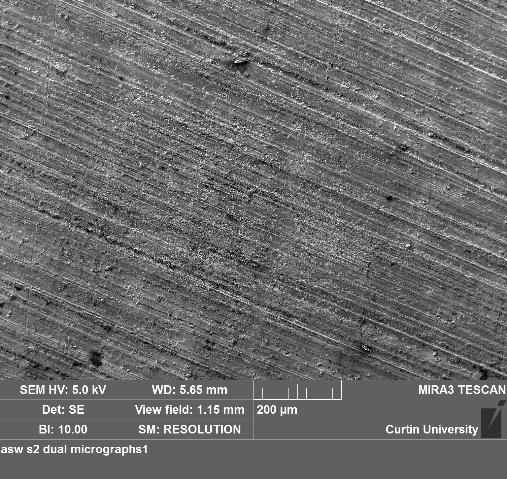b) K. pneumoniae* on wet-ground surface after biocide application | *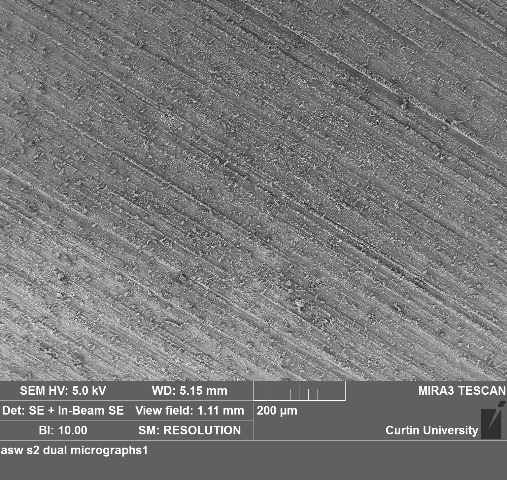c) P. balearica* on wet-ground surface after biocide application |
| --- | --- | --- |
| *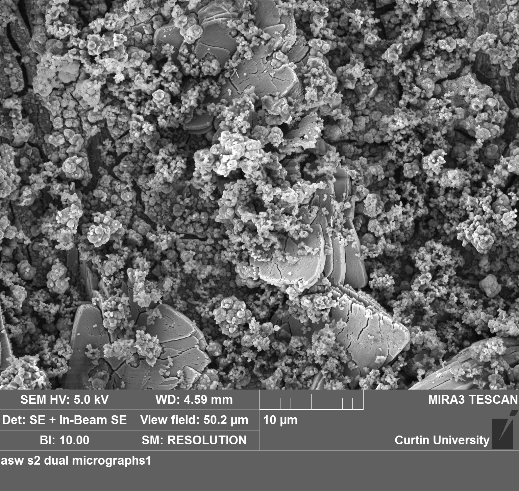d) S. chilikensis* on oxidised surface after biocide application | *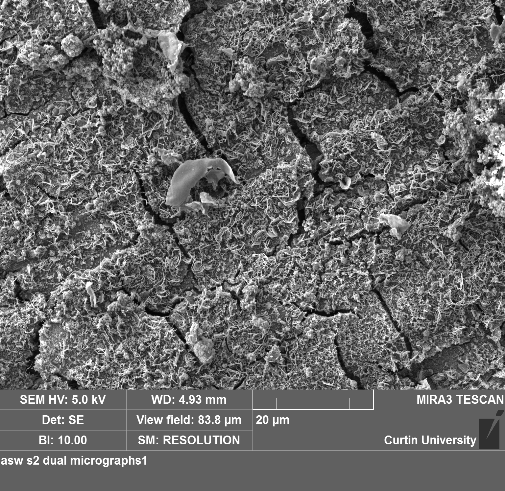e) K. pneumoniae* on oxidised surface after biocide application | *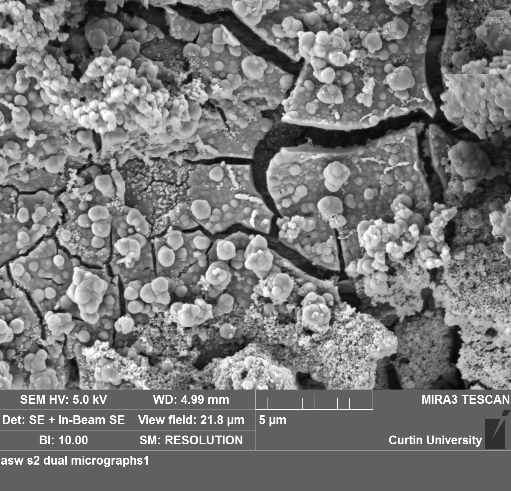f) P. balearica* on oxidised surface after biocide application (showing cells) |

***Supplementary Figure 3:*** *SEM micrographs of wet ground and oxidised surfaces after inhibitor application.*

Confocal Raman Spectroscopy


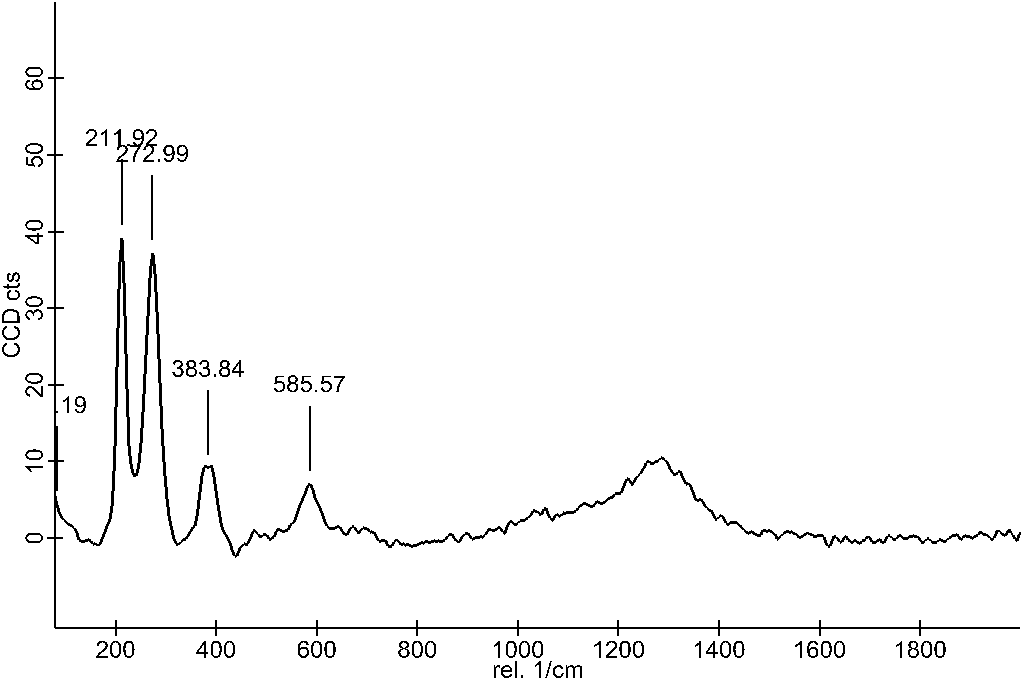


***Supplementary Figure 4:*** Representative confocal Raman spectra of oxidised surface with 4 major peaks (212, 272, 384 and 586 rel. 1/cm).
